# Determination of the Degree of Degradation of Frying Rapeseed Oil Using Fourier-Transform Infrared Spectroscopy Combined with Partial Least-Squares Regression

**DOI:** 10.1155/2015/185367

**Published:** 2015-02-23

**Authors:** Jie Yu Chen, Han Zhang, Jinkui Ma, Tomohiro Tuchiya, Yelian Miao

**Affiliations:** ^1^Faculty of Bioresource Sciences, Akita Prefectural University, Akita 010-0195, Japan; ^2^School of Chemistry & Chemical Engineering, Zhaoqing University, Zhaoqing 526061, China; ^3^College of Food and Light Industrial Engineering, Nanjing University of Technology, Nanjing 211800, China

## Abstract

This rapid method for determining the degree of degradation of frying rapeseed oils uses Fourier-transform infrared (FTIR) spectroscopy combined with partial least-squares (PLS) regression. One hundred and fifty-six frying oil samples that degraded to different degrees by frying potatoes were scanned by an FTIR spectrometer using attenuated total reflectance (ATR). PLS regression with full cross validation was used for the prediction of acid value (AV) and total polar compounds (TPC) based on raw, first, and second derivative FTIR spectra (4000–650 cm^−1^). The precise calibration model based on the second derivative FTIR spectra shows that the coefficients of determination for calibration (*R* ^2^) and standard errors of cross validation (SECV) were 0.99 and 0.16 mg KOH/g and 0.98 and 1.17% for AV and TPC, respectively. The accuracy of the calibration model, tested using the validation set, yielded standard errors of prediction (SEP) of 0.16 mg KOH/g and 1.10% for AV and TPC, respectively. Therefore, the degradation of frying oils can be accurately measured using FTIR spectroscopy combined with PLS regression.

## 1. Introduction

The quality of fried foods is closely related to the quality of the frying oil [1]. During frying, oil is subjected to prolonged periods of heating at high temperatures in the presence of air and water. This leads to a wide range of complex chemical reactions, such as thermal oxidation, hydrolysis, and polymerization [2, 3]. The compounds generated by these chemical reactions not only adversely affect flavor but also produce undesirable constituents in fried foods [4]. Therefore, controlling frying oil quality by a rapid evaluation method is imperative. Several quality attributes are used to evaluate the quality of frying oil by traditional chemical methods, acid value (AV) or free fatty acids (FFA), total polar compounds (TPC), or total polar materials (TPM). Most standard analytical methods for oil analysis are expensive, require lengthy sample preparation, and, in some cases, depend on advanced instruments [5–7]. Thus, physical methods, based on colorimetric reaction, refractive index, density, or viscosity [5, 8, 9], are relatively easy for measurement, but do not agree well with the chemical changes in frying oils during deep-fat frying.

Fourier-transform infrared (FTIR) spectroscopy has proved to be a viable alternative to standard wet analytical techniques for determining key quality parameters in many fields [10–12]. Previous studies have identified many adulteration problems using experimental and statistical methods [13–17]. More recently, FTIR spectroscopy was used to determine deterioration in edible vegetable oils adulterated with used frying oil [18, 19]. These investigations highlighted the use of FTIR spectroscopy to authenticate and monitor edible vegetable oils but did not evaluate frying oils that have actually been used to fry foods. Al-Degs et al. [20] used FTIR spectroscopy and a multivariate calibration method to measure the degradation of frying quality of used vegetable oils collected from 25 public restaurants. Relatively high correlations were reported between results from wet chemical analysis and infrared spectroscopy, but the number of the samples and the measurement ranges were limited.

Du et al. [21] discussed evaluating the quality of deep frying oils with FTIR spectroscopy using a large number of deep frying oil samples collected from a local college canteen, but only the FFA and peroxide values (PV) of frying oils were examined. As it is well known, the PV value indicates the degree of oxidation of oils in the natural state or in the initial stages of oxidation, so it cannot be used for evaluating the quality of deep frying oils. The FFA value indicates the quantity of free fatty acids by hydrolysis, so again it cannot be used for evaluating the quantity of secondary oxidation compounds that are connected to the quality of deep frying oils. The TPC value is the most important parameter for evaluating the quality of frying oils, because it indicates the quantity of secondary oxidation compounds. Shen et al. [22] used FTIR spectroscopy to assess the quality (FFA and TPC values) of deep frying oils used by street vendors but no frying rapeseed oil samples were used.

The objective of the present study was to investigate whether the AV and TPC values of frying rapeseed oil samples that degraded to different extents could be determined by FTIR spectroscopy combined with partial least-squares regression in order to determine the degree of degradation of a large number of deep frying oils. Furthermore, it will also be determined whether the derivative spectra could improve the measurement accuracy of the model.

## 2. Experimental

### 2.1. Sample Preparation

Two types of rapeseed oil (commercially refined canola oil from the Nisshin Oillio Group Ltd., Tokyo, Japan, and unrefined rapeseed Kizakinonatane oil from Akita New Bio Farm Co., Ltd., Akita, Japan) were used as the frying oil samples. Frozen par-fried French fries in an institutional pack were purchased from a local supermarket and used for deep frying. The frying was conducted in a restaurant-style stainless steel electric fryer TF-40A (Taiji & Co., Ltd., Kanagawa, Japan) at frying temperatures of 180, 200, or 220°C. Batches of 100 g of frozen French fries were fried for 3 min at 22 min intervals, when the temperature of the oil had reached the desired temperature. This continued for a period of 7 h each day for 4 consecutive days. This was equivalent to frying 17 batches per day and therefore 68 batches for the whole experiment. During the frying process, 200 mL of heated oil was drawn off every 3.5 h and stored at −18°C until analysis for AV and TPC values and the acquisition of NIR spectral data. The frying experiments were carried out once using canola oil and twice using rapeseed Kizakinonatane oil. A total of 156 frying oil samples, degraded to different degrees, were obtained from the food frying process.

### 2.2. Reference Analysis

The acid values (AV) of the frying oil samples were determined in triplicate using an automatic potentiometric titrator (AT-500N, Kyoto Electronics Manufacturing, Kyoto, Japan) according to the AOCS Official Method Cd 3d-63 [23]. All AV analysis results were expressed as mg KOH/g oil. The total polar compound (TPC) estimations of the frying oil samples were measured directly with a food oil monitor (FOM 310, Ebro Electronics, Ingolstadt, Germany) based on measuring the change in dielectric constant.

### 2.3. FTIR Spectral Acquisition

Spectroscopic data from the frying oil samples were acquired by an infrared spectrometer (Nicolet 6700 FT-IR, Thermo Fisher Scientific K.K, Waltham, MA, USA) equipped with an attenuated total reflection (ATR) accessory with a temperature controller. A small amount of the oil samples was uniformly deposited on the crystal surface of the ATR accessory (Specac Inc., Woodstock, GA, USA), equipped with a ZnSe reflection crystal. Analyses were carried out at room temperature. Spectra were acquired (100 scans/sample or background) in the wavenumber range of 4000–650 cm^−1^ at a spectral resolution of 4 cm^−1^, and the data was exported using OPUS Ver. 6.0 (Bruker Optics, Billerica, MA, USA) software in ASCII-compatible format. For each sample, the absorbance spectrum was collected against a background obtained with a dry and empty ATR cell. Three spectra per sample were recorded. After acquiring each spectrum, the ATR crystal was cleaned with a cellulose tissue soaked in hexane and then rinsed with acetone. All spectral data exported by the OPUS software were then imported to Unscrambler software (CAMO, Oslo, Norway).

Because there are differences in the viscosity of frying oil samples, it may produce baseline offset and slope in infrared spectra that may affect the creation of robust calibration model. One of the earliest methods for removing baseline offset and slope is the use of derivative spectra [24, 25]. The first derivative of a spectrum is simply a measure of the slope of the spectral curve at every point. The slope of the curve is not affected by baseline offsets in the spectrum, and thus the first derivative is a very effective method for removing baseline offsets. The second derivative is a measure of the change in the slope of the curve. In addition to ignoring the offset, it is not affected by any linear “tilt” that may exist in the data and is therefore a very effective method for removing both the baseline offset and slope from a spectrum. The first and second derivative spectra were obtained using the Savitzky-Golay method [26] with a segment of a 10-point window and a gap of a 0-point window. The derivative mathematical treatments were carried out using the Unscrambler software.

### 2.4. Statistical Analysis

Partial least-squares (PLS) regression analysis was used to extract relevant information from the complex FTIR spectra of the frying oils. The calibration models were created by PLS regression from the raw FTIR spectra and their first and second derivatives, and the optimum number of PLS factors used for prediction was determined by full cross validation. The number of significant PLS factors was chosen using a predicted residual error sum of squares (SECV) value for every possible factor. The SECV value was the sum of the squared differences between the predicted and known concentrations. It was calculated by building calibration models with different numbers of factors and then predicting some samples of known concentration against the model. The quality of the calibration model is described by the squared correlation coefficient (*R* ^2^) and standard error of calibration (SEC) and was validated with the validation sample set that was not used for the calibration development. The correlation coefficient of prediction (*r*) and root-mean square of the prediction (SEP) were used to choose the best model. SEP measures how well the model can predict samples in a validation set. The best calibration model to be used for prediction was the one with the highest value of *r* and the lowest value for SEP. The PLS regression performance was carried out using PLS1 algorithm with only one *Y*-variable for prediction of the AV or TPC value of frying rapeseed oil samples, respectively, by the Unscrambler software.

The 156 oil samples obtained from the frying process were divided into calibration and validation sets as follows. Initially, samples within the parent set were sorted according to AV or TPC values determined by traditional methods. Starting with the sample with the lowest AV or TPC value, the first and third samples were assigned to the calibration set and the second sample to the validation set. The next group of three samples was assigned similarly, and so on, until the last group. Thus, 104 samples were included in the calibration set and 52 in the validation set. Statistics for the AV and TPC values of the frying oil samples selected for the calibration and validation sets are shown in Table 1.

## 3. Results and Discussion

### 3.1. Spectra Features of FTIR

**Figure 1:**
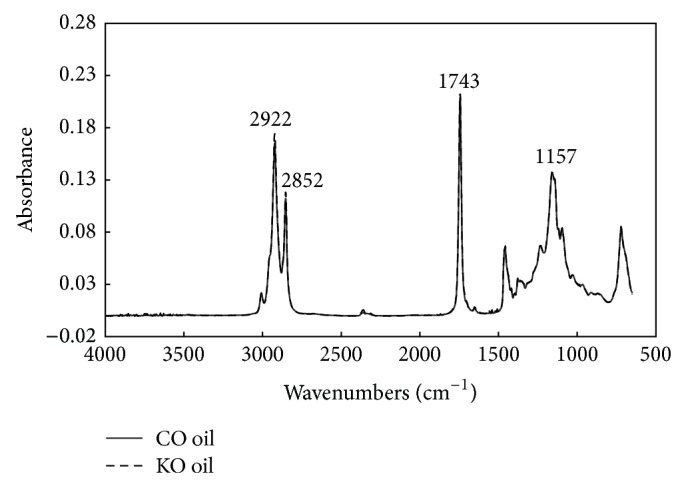
FTIR spectra of canola (CO) and Kizakinonatane (KO) frying oil samples.

Figure 1 shows the FTIR spectra of fresh canola oil and rapeseed Kizakinonatane oil. To the naked eye, the entire ranges of spectra of the two oils look similar. These spectra showed the typical characteristic of absorption bands similar to those reported in previous studies [15, 16, 20, 27]. The most prominent absorption band at 1743 cm^−1^ can be assigned to the C=O stretching of aliphatic esters [15, 16, 20, 27]. The strong bands at around 2922 and 2852 cm^−1^ can be ascribed to the asymmetrical and symmetrical C-H stretching vibrations of CH_2_ groups. The band at around 1157 cm^−1^ can be assigned to the stretching of the C-O bonds of aliphatic esters [15, 16, 20, 27].

**Figure 2:**
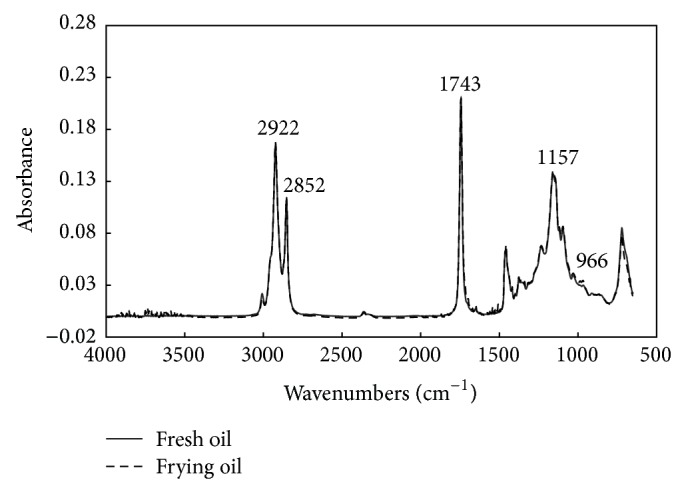
FTIR spectra of the fresh and used canola oil.

Figure 2 shows the FTIR spectra of fresh and used canola oil. The entire range of spectra looks very similar for the fresh (before frying) and used oils (after frying). However, if one examines the spectra closely, differences between fresh and used canola oil samples are observed at the typical characteristic of absorption bands around 2922, 2852, 1743, and 1157 cm^−1^. In addition, the weak absorption at 966 cm^−1^ observed in used oil samples may be due to the C-H out-of-plane deformation of isolated* trans* double bonds or some* trans* conjugated unsaturated fatty acids [15, 16, 20, 27, 28]. There was an obvious difference in absorption intensity at around 966 cm^−1^ between the fresh (before frying) and the used oils (after frying). The peak intensity at 966 cm^−1^ exhibited a slight increase in used frying oils compared with the fresh oil. Thus, it can be seen that much information about the degradation of frying oils can be obtained from the entire range of FTIR spectra.

### 3.2. IR Calibration Models for AV and TPC Values

PLS regression analysis results for predicting AV and TPC values in frying oils using raw, first, and second FTIR spectra are shown in Table 2. A total of six PLS calibration models were developed for analyzing the AV and TPC value of frying oils using the calibration and validation sample sets based on raw, first, and second derivative spectra. There were very strong correlations between actual values and IR-predicted values in these calibration models, with *R* ^2^ values 0.99 for AV and 0.98 for TPC.

When the SEC, SECV, and SEP values of the calibration models were compared as shown in Table 2, the results based on the first or second derivative spectra were better than those based on the raw spectra. The baseline offset and slopes in spectra might affect the development of a sensitive calibration model because of the differences in the viscosity of the frying oil samples. The first derivative of the raw spectra is simply a measure of the slope of the spectral curve at every point and the second derivative is a measure of the change in the slope of the curve. The slope of the curve is not affected by baseline offsets in the spectrum, and thus using the first and second derivatives is a very effective method for removing baseline offsets [24, 29, 30]. This may be why relatively better results were obtained based on the first or second derivative spectra.

**Figure 3:**
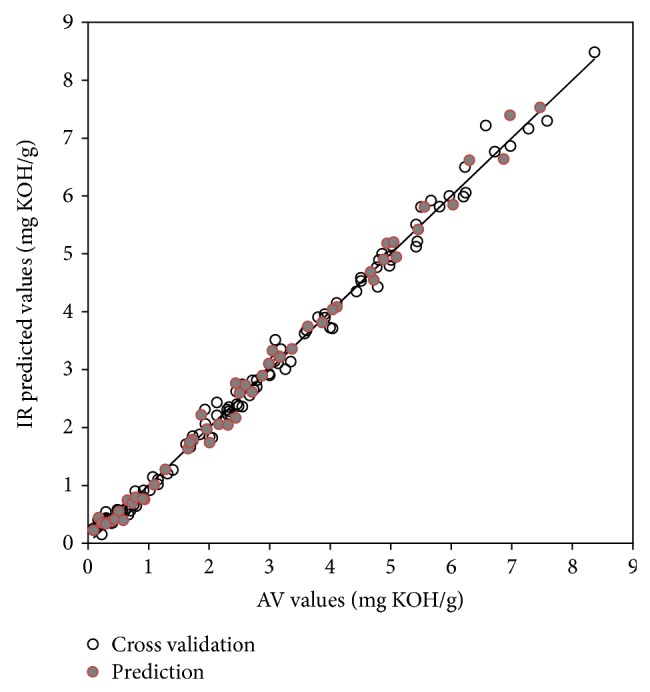
Relationship between actual and IR-predicted AV values.

**Figure 4:**
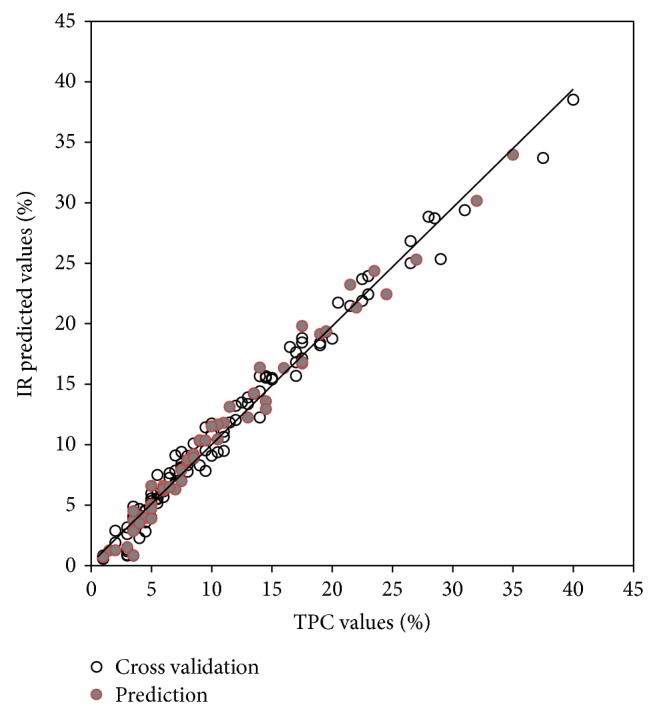
Relationship between actual and IR-predicted TPC values.

The calibration models based on the first and second derivative spectra gave the same level of accuracy. Because the wavelengths of the absorbing peaks are in accordance, despite the fact that second derivative spectrum and the raw spectrum are being complete opposite of direction of the absorption peaks, the models based on the second derivative spectra were chosen in order to easily examine the factors contributing to these models, which for AV showed low values of SECV (0.16 mg KOH g^−1^) and SEP (0.16 mg KOH g^−1^) and for TPC and low values of SECV (1.17%) and SEP (1.10%). Compared with the results of AV in previous studies [20–22], higher *R* ^2^ and lower SECV and SEP values were obtained. The differences between previous studies and the present study were mainly due to the wider range of frying oil samples and the differences in the numbers of samples. The cross validation and prediction (validation) results, represented graphically by plotting the reference analysis values against the IR-predicted values based on the second derivative spectra, showed strong linearity, as shown in Figures 3 and 4. Furthermore, all the prediction models had high ratio performance deviation (RPD) values of more than 5.8. The RPD is the ratio between the standard deviation of the observed values of training samples against the SEP of the model; the larger it is, the better the technique does perform. Generally, an RPD value above 3 indicates a useful model that allows good quantitative predictions [31–33]. It can thus be concluded that the IR spectra provided good estimates of AV and TPC values in frying oils, showing low SEP values and very high RPD values.

### 3.3. Regression Coefficients of PLS Model

Regression coefficients can be used to compare the contributions of individual wavenumbers to a PLS calibration model, since a regression coefficient spectrum shows characteristic peaks and troughs that can indicate which wavenumber range is important for the calibration model [29, 32, 34].

**Figure 5:**
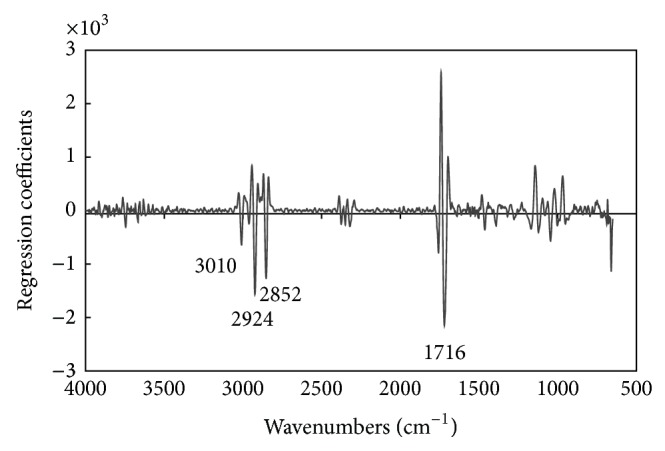
Regression coefficients of the PLS calibration model for AV based on the second derivative spectra.

Figure 5 shows the regression coefficients of the PLS calibration model based on the second derivative spectra for AV values. Some notable negative peaks at wavenumbers 3010, 2924, 2852, and 1716 cm^−1^ were easily observed. The negative peak at a wavenumber of 1716 cm^−1^ can be assigned to the C=O functional group, which might be related to the absorption of the C=O stretching characteristic frequency associated with free fatty acids that increase with the degradation of frying oils [14, 19, 35, 36]. The negative peaks at wavenumbers 3010, 2924, and 2852 cm^−1^ can be assigned to the C-H functional group, which might be related to the absorption of the C-H stretching characteristic frequency associated with free fatty acids [14, 19, 35, 36].

**Figure 6:**
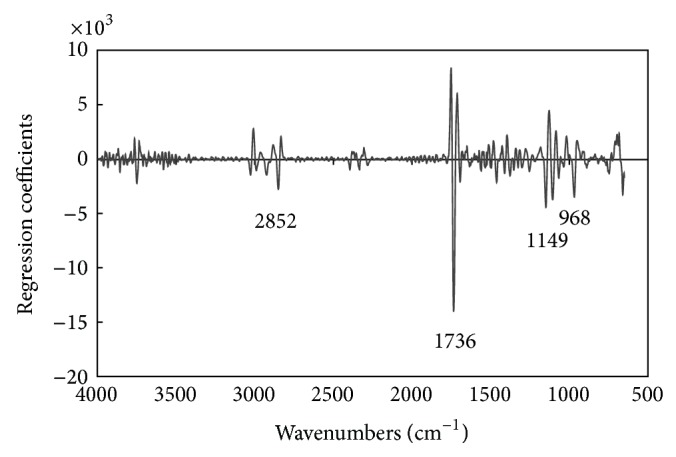
Regression coefficients of the PLS calibration model for TPC based on the second derivative spectra.

Figure 6 shows the regression coefficients of the PLS calibration model based on the second derivative spectra for TPC values. Some notable peaks at wavenumbers 3026, 3008, 2852, 1736, 1149, and 968 cm^−1^ were easily observed. The negative peak at wavenumber 1736 cm^−1^ can be assigned to the C=O functional group, which might be related to the absorption of the C=O stretching characteristic frequency associated with ester, aldehyde, and ketone, which increase with the degradation of frying oils [14, 19, 35, 36]. The negative peak at wavenumber 3026 cm^−1^ can be assigned to the C-H* trans* functional group and the positive peak at wavenumber 3008 cm^−1^ can be assigned to the C-H* cis* functional group, which might be related to unsaturated fatty acids that increase with* trans* fatty acids and decrease with* cis* fatty acids in frying oils [14, 19, 35, 36]. The negative peak at wavenumber 968 cm^−1^ can be assigned to the HC=CH* trans* bending (out-of-plane) functional group, which might be related to the absorption of* trans* fatty acids that increase with the degradation of frying oils [14, 19, 35, 36]. Taken together, these results suggest that PLS calibration models with very high precision for prediction of AV and TPC values of frying rapeseed oils could be established. These are based on the absorption of FFA and secondary oxidation products such as carboxylic acid, ester, aldehyde, and ketone groups, which all increase as frying oils deteriorate. The results of the present study have thus shown the utility of using the IR technique to determine the AV and TPC values of frying oils.

## 4. Conclusions

FTIR spectroscopy can be successfully applied to measure the AV and TPC values of frying rapeseed oils with high precision. Good calibration model based on the second derivative spectra was obtained by comparing the prediction accuracy of models based on raw, first, and second derivative spectral data. FTIR spectroscopy has significant advantages over chemical analytical techniques; it is a fast and simple method that requires no sample preparation, so it is a very practical method for measuring the AV and TPC values of frying oils.

## Figures and Tables

**Table 1:** Characteristics of reference data for calibration and validation sets.

	Calibration set (*n* = 104)			Validation set (*n* = 52)		
	Mean	Range	SD	Mean	Range	SD
AV (mg KOH g^−1^)	2.82	0.09–8.37	2.06	2.80	0.09–7.47	2.02
TPC (%)	11.00	0.50–40.00	8.21	10.92	0.50–35.00	7.97

SD: standard deviation.

**Table 2:** PLS analysis results for predicting the AV and TPC values of frying oil samples.

		*F*	*R* ^2^_cal_	SEC	SECV	*R* ^2^_val_	SEP	Bias	RPD
AV	Raw spectra	5	0.99	0.20	0.22	0.99	0.22	0.03	9.2
	First derivative spectra	5	0.99	0.15	0.17	0.99	0.16	0.03	13.0
	Second derivative spectra	6	0.99	0.14	0.16	0.99	0.16	0.03	12.7

TPC	Raw spectra	6	0.98	1.13	1.26	0.98	1.13	0.00	7.1
	First derivative spectra	6	0.98	1.04	1.16	0.98	1.10	−0.09	7.3
	Second derivative spectra	6	0.98	1.05	1.17	0.98	1.10	−0.01	7.3

*F*: number of factors; *R* ^2^: coefficient of determination; SEC: standard error of calibration; SECV: standard error of cross validation; SEP: standard error of prediction; bias: average of differences between reference value and NIR value; RPD: ratio of standard deviation of reference data in the validation set to SEP.
